# Public Attitudes to Digital Health Research Repositories: Cross-sectional International Survey

**DOI:** 10.2196/31294

**Published:** 2021-10-29

**Authors:** Giovanna Nunes Vilaza, David Coyle, Jakob Eyvind Bardram

**Affiliations:** 1 Department of Health Technology Technical University of Denmark Kongens Lyngby Denmark; 2 School of Computer Science University College Dublin Dublin Ireland

**Keywords:** digital medicine, health informatics, health data repositories, personal sensing, technology acceptance, willingness to share data, human-centered computing, ethics

## Abstract

**Background:**

Digital health research repositories propose sharing longitudinal streams of health records and personal sensing data between multiple projects and researchers. Motivated by the prospect of personalizing patient care (precision medicine), these initiatives demand broad public acceptance and large numbers of data contributors, both of which are challenging.

**Objective:**

This study investigates public attitudes toward possibly contributing to digital health research repositories to identify factors for their acceptance and to inform future developments.

**Methods:**

A cross-sectional online survey was conducted from March 2020 to December 2020. Because of the funded project scope and a multicenter collaboration, study recruitment targeted young adults in Denmark and Brazil, allowing an analysis of the differences between 2 very contrasting national contexts. Through closed-ended questions, the survey examined participants’ willingness to share different data types, data access preferences, reasons for concern, and motivations to contribute. The survey also collected information about participants’ demographics, level of interest in health topics, previous participation in health research, awareness of examples of existing research data repositories, and current attitudes about digital health research repositories. Data analysis consisted of descriptive frequency measures and statistical inferences (bivariate associations and logistic regressions).

**Results:**

The sample comprises 1017 respondents living in Brazil (1017/1600, 63.56%) and 583 in Denmark (583/1600, 36.44%). The demographics do not differ substantially between participants of these countries. The majority is aged between 18 and 27 years (933/1600, 58.31%), is highly educated (992/1600, 62.00%), uses smartphones (1562/1600, 97.63%), and is in good health (1407/1600, 87.94%). The analysis shows a vast majority were very motivated by helping future patients (1366/1600, 85.38%) and researchers (1253/1600, 78.31%), yet very concerned about unethical projects (1219/1600, 76.19%), profit making without consent (1096/1600, 68.50%), and cyberattacks (1055/1600, 65.94%). Participants’ willingness to share data is lower when sharing personal sensing data, such as the content of calls and texts (1206/1600, 75.38%), in contrast to more traditional health research information. Only 13.44% (215/1600) find it desirable to grant data access to private companies, and most would like to stay informed about which projects use their data (1334/1600, 83.38%) and control future data access (1181/1600, 73.81%). Findings indicate that favorable attitudes toward digital health research repositories are related to a personal interest in health topics (odds ratio [OR] 1.49, 95% CI 1.10-2.02; *P*=.01), previous participation in health research studies (OR 1.70, 95% CI 1.24-2.35; *P*=.001), and awareness of examples of research repositories (OR 2.78, 95% CI 1.83-4.38; *P*<.001).

**Conclusions:**

This study reveals essential factors for acceptance and willingness to share personal data with digital health research repositories. Implications include the importance of being more transparent about the goals and beneficiaries of research projects using and re-using data from repositories, providing participants with greater autonomy for choosing who gets access to which parts of their data, and raising public awareness of the benefits of data sharing for research. In addition, future developments should engage with and reduce risks for those unwilling to participate.

## Introduction

### Background

Health research is increasingly adopting digital technologies to accelerate scientific discovery, as digital data sources increase scalability and predictive power for algorithmic inferences [1-3]. Novel data collection techniques include wearables and smartphone sensors to extract participants’ behavioral features passively [4]. Records of calls and texts can flag social activity fluctuations; location tracking can reveal mobility patterns; heart rate measures can indicate sleep quality [5-7]. Ecological momentary assessments further complement such passive indicators by sampling individuals’ health status in real time through questionnaires [8,9]. The motivation for considering pervasive and digital sources of health and behavioral information is related to the possibility of closely observing research patients’ daily lives [10,11].

Intending to personalize future patient care, researchers search for scientific evidence by analyzing longitudinal streams of personal sensing data from large segments of the population [12,13]. Because of this expansion of personal sensing in the health domain, digital health research repositories are gaining momentum. An ambitious concept similar to biobanks [14], a digital health research repository allows multiple projects and researchers to share access to personal data streams beyond DNA and biosamples [15-17]. Although still in their initial steps, initiatives with this goal in mind include nationwide programs [18,19], university-led projects [20,21], and community-driven data platforms [22-24].

Despite promising benefits, barriers to public acceptance can hinder the successful implementation of digital health research repositories [25,26]. Without a diverse range of participants agreeing to contribute with their personal health data, repositories cannot accomplish their ambitious goal of providing reliable evidence for personalized medicine to the broad population [27]. Furthermore, a potential lack of acceptance is aggravated by ethical debates questioning which rights individuals should have following contribution of their data [28-30], especially if data are shared beyond a specific project’s scope. Given such challenges, previous research has emphasized that in contrast to most current initiatives, which mainly cater to researchers’ needs, health data repositories should attend more to participants’ preferences to identify enablers for participation [31,32].

### Previous Studies

Previous studies have investigated public attitudes toward biobanks [33-36] and digital health data [37,38] separately. Other past studies have examined motivations to contribute [39], privacy concerns [40], and access control preferences [41] for data sharing within health research in general, yet these studies consider only a few variables simultaneously and rarely inquire about the magnitude of specific attitudes [42,43]. To the best of our knowledge, published quantitative studies have not thoroughly examined how different factors can affect attitude and willingness to share in digital health research repositories’ timely and emerging context. Thus, it remains unclear how the public perceives the risks and benefits of shared access of multiple sources of behavioral and health indicators, including digital sensing, for research repositories.

### Study Goal

Given this research gap, an online cross-sectional survey was conducted examining public attitudes to research repositories storing health information, biosamples, personal sensing, and behavioral data. This survey study aims to identify implications for future developments by consulting those whose personal data are to be shared for research. The study took place in Denmark, where the project is funded, and the principal investigators are based. Furthermore, with the goal of investigating the potential contrast between 2 very different historical, social, and cultural contexts, we contacted a research group in Brazil to establish a partnership and conduct the study with a sample of Brazilian residents. This decision allowed a cross-country analysis that illuminated similarities and divergences between 2 very disparate contexts. The results contribute to substantial empirical evidence about enablers and barriers for participants’ acceptance and discussions on how community engagement, technology design, and policymaking can lead to a stronger participant-centric development in this field.

## Methods

### Population, Sample, and Recruitment

Denmark and Brazil are very different in terms of population, geography, economy, and culture. Denmark is a small country in area and population (5.8 million inhabitants), with a robust economy and a strong focus on social welfare, which is evident given the country’s investments in education, research, and health care. By contrast, Brazil, the fifth largest country globally (208 million inhabitants), has a diversified economy, rich biodiversity, and industrial potential but at the same is characterized by an unequal society. Most of the population still lacks access to high-quality education and health services given the vast disparities of wealth distribution across the country. These differences make the population of these 2 countries very contrasting.

This survey study was part of an academic consortium project, publicly funded, to develop a digital health research repository for youth mental health, in turn defining our main target population (young adults), but without excluding the possibility of collecting data from other, older groups, if those participants would be interested in the study. As the project investigators are in Denmark, participants were first recruited among young Danish residents. Later, to enable the comparison of findings with a divergent historical, cultural, and social context, we sought to form a partnership with clinical researchers at a university in Northeast Brazil, the Universidade Federal do Rio Grande do Norte (UFRN). Therefore, recruitment efforts were also made to collect data from a sample of young adults in Brazil, but without being restrictive over the age groups that could respond. The online survey was deployed using LimeSurvey and hosted on a server at the Technical University of Denmark (DTU). The survey link was distributed across several channels in an extensive recruitment process that started on March 9, 2020, and ended on December 9, 2020. The link was made available in forums and newsletters at university portals, emailing lists, social networking groups, online chat platforms, and unpaid posts on Twitter. Recruitment in person happened once during an event hosted at DTU (prior to the surge of COVID-19). Participants were compensated with a cup of coffee at this event. Besides this one-time event, no other compensation was given to respondents to avoid providing incentives for repeated participation. Given the distribution of the survey in multiple channels without access restriction, a considerable number of responses came from participants beyond the target population (older than 27 years old). The research team decided not to exclude data from these respondents belonging to age groups above 27 years from analysis; instead, the data collected enabled another dimension for comparison (age). The sample, therefore, includes participants from all age groups residing in Brazil and Denmark.

### Ethical and Legal Compliance

Following local jurisdictions, this survey study received ethical approval from the Institutional Review Board of the partner university in Northeast Brazil and was exempt from ethical approval in Denmark. As established by the European General Data Protection Regulation (GDPR), the first page of the survey included information about the study’s purpose, which data were collected, measures to anonymity and confidentiality, and data handling processes. Participants were asked to provide their consent after reading this information and confirming that they were older than 18 years. Besides the consent question, none of the questions were mandatory, following standard ethical conduct principles for online research. In addition, the survey was anonymous (IP address and identifiable information were not collected).

### Questionnaire Design

#### Overview

The instrument development was based on (1) several previous surveys and focus groups about public acceptance of biobanks, electronic health records, and clinical trial repositories [43-53]; (2) a previous qualitative study about enablers and barriers for participation in digital health research repositories [32]; and (3) the input from the research team, clinicians, statisticians, and participants of the target population (young adults). The instrument went through several iterations until the final version, which is the one available in Multimedia Appendix 1. First, questions were designed in English and this version was used to ask for the initial rounds of feedback from both experts and targeted participants. After each round of feedback, the questionnaire was incrementally modified. Once a final English version was agreed upon by the research team, the questionnaire was professionally translated to the official language of each country (Portuguese and Danish). The translations were then verified by native speakers from the research team (GV and JB) to ensure content validity. Using the translated versions and the original in English, pilot tests were conducted by the research team with small convenient samples of 5 young adults in Denmark (in person) and in Brazil (remotely). These pilot tests consisted of asking participants to fill the survey and provide feedback on the readability of the questions, comprehension of the vocabulary used for the answer options, navigation of the interface, and time taken to complete all questions. Participants unanimously expressed that the instrument was easy to use and understand and completion time was reasonable. All members of the research team then approved the distribution of the final versions of the questionnaire (in Portuguese, English, and Danish). The complete questionnaire is available in Multimedia Appendix 1. A summary of the survey questions and their rationale are described next. This study is the first to combine such a set of diverse factors to the best of our knowledge.

#### Demographics and Socioeconomics Questions

This first group of questions inquired about participant age group, gender, education level, country of residence, and usage and ownership of digital devices (computers, smartphones, smartwatches, smart home assistants, and tablets). Some individuals may have a gender that is neither male nor female. They may identify as both male and female at one time, different genders at different times, no gender at all, or dispute the very idea of only 2 genders. Therefore, the term “nonbinary” in this study refers to gender identities outside of the gender binary (male or female). The survey conducted in Brazil also contained 2 additional questions: race and household income (based on minimum salary). Minimum salary has been defined as the minimum amount of remuneration that an employer is required to pay for the work performed during a given period (usually per month), which cannot be reduced by collective agreement or an individual contract. In Brazil, at the time of the study, the minimum salary per month was 1040 Brazilian Reais (approximately US $188.45). Following recommendations by local Brazilian investigators, these questions were added to examine whether the sample reflected the Brazilian population’s diversity, which, by contrast, was not considered a usual requirement by local investigators in Denmark.

#### Factors Related to Technology Acceptance

This group of questions asked participants about factors highlighted by previous research as essential for technology acceptance in data-sharing contexts: self-assessed health status [54], personal interest in health topics [34], previous participation in health research [33], and awareness of examples of data repositories [55]. This group of questions also asked participants about their current attitude toward digital health research repositories (positive, negative, or indifferent) [43,56] after being provided with the following short description of the concept: A research data repository is an online database containing data collected during research studies. In such repositories, deidentified data is to be re-used in the future by other research studies.

#### Motivations to Participate and Reasons for Concern

These questions asked participants how motivated they would feel by the following reasons to contribute to a research data repository: helping future patients, helping researchers, receiving results about themselves, knowing the research outcomes, getting financial compensation, and proposing questions to be investigated in future studies. Participants were also asked how concerned they would feel about the following risks if their data were stored in a health research repository: having their data used for profit without their knowledge, having data used for projects that they perceive as unethical, agreeing with terms and conditions that they do not fully understand, being socially discriminated against because of the information shared, becoming vulnerable to cyberattacks and blackmail, and being asked to provide more data in the future. Such questions about motivations and concerns were based on findings of a qualitative interview study [32] and previous research on motivations to contribute to research [57] and concerns related to data sharing in general [58,59]. The order of the answer options was randomized for each respondent to avoid order bias.

#### Access Control Preferences

This group of questions asked participants how desirable or undesirable different access control choices would be once they shared their data with a research platform (answers were not mutually exclusive). The listed answers were: to never be contacted after data are shared, to receive information about who is using the data, to decide who has access to which parts of the data, to have the repository managers decide who has access, to grant data access to public or academic institutions, and to give data access to private laboratories and companies. These questions were based on previous research about informed consent options in biobanks and health data–sharing contexts [48,51,58,60]. The order of the answer options was randomized for each respondent to avoid order bias.

#### Willingness to Share Data

Questions in this group concerned how comfortable or uncomfortable participants would feel about sharing different deidentified data sources for a research repository, as previous studies have shown that willingness to share personal health data varies according to the data source [61-63]. Data sources were grouped as (1) biospecimen samples and input data provided through health questionnaires (online or in-person); and (2) passive data collected through smartphone or wearable devices, without end user input. The first questions inquired about participants’ willingness to share the following: clinical diagnosis (physical), clinical diagnosis (mental), family health status, DNA samples, food consumption, alcohol consumption, sleep patterns, and blood samples. These data types were based on previous studies of willingness to share clinical and health data for research [64]. The second group of questions inquired about participants’ willingness to share frequency of social communication (calls/texts), the content of social communication (calls/texts), distances traveled per day, places visited, physical activity levels (heart rate), stress/emotional levels (heart rate), screen time, and apps used. The choice of data types to include in this second group was based on digital data sources previously identified as objective behavioral features for health research [5]. Based on previous studies that showed that different granularities might affect willingness to share, the options in this second group were purposely varied in terms of levels of detail provided by the sensor data (eg, frequency of calls/texts versus the content of calls/texts) [65]. The order of the answer options was randomized for each respondent to avoid order bias.

### Statistical Analysis

Data were analyzed and visualized using the R Project for Statistical Computing (software environment for statistical computing and graphics). First, frequency distributions were used to characterize responses for each variable, and bivariate associations (odds ratio [OR]) examined relationships between variables. Following previously established reference values, an OR below 1.5 was considered weak and above 5.0 strong [66]. For a 95% CI, results were considered significant if *P*<.05. Then, a binary logistic regression was conducted to examine directional relationships between explanatory variables and participants’ current attitudes toward digital health research repositories. Similarly, another binary logistic regression was conducted to examine directional relationships between explanatory variables and participants’ willingness to share data types. Missing values from “prefer not to say” responses were removed before conducting these regression analyses and assumptions were verified beforehand.

## Results

### Survey Participants

A total of 2299 participants started answering the survey, of whom 1963 completed all questions (1963/2299, 85.38%). This paper includes only responses from participants living in Denmark (583/1600, 36.44%) and Brazil (1017/1600, 63.56%), thus excluding participants residing in other countries from the data analysis for this study (336/1963, 17.12%). The majority of the sample is aged between 18 and 27 years (933/1600, 58.31%); the second largest age group is between 28 and 37 years (459/1600, 28.69%). Only 12.56% (201/1600) were aged above 37 years. There are slightly more individuals who identify as females (891/1600, 55.69%) than males (682/1600, 42.63%). A majority of participants are educated, having at least a university degree (992/1600, 62.00%), own and use smartphones (1562/1600, 97.63%) and computers (1537/1600, 96.06%), but only 36.75% (588/1600) own and use more than 2 types of digital devices. The vast majority is currently in good, very good, or excellent health (1407/1600, 87.94%), while most are moderately, very, or extremely interested in health topics (1088/1600, 68.00%). Around half of the Brazilian participants (555/1017, 54.57%) are White and 43.17% (439/1017) are Black or Brown; most of the respondents living in Brazil have a monthly household income between 1 (1040 Brazilian Reais or US $190) and 5 (5200 Brazilian Reais or US $950) minimum salaries (739/1017, 72.66%). As explained in the previous section, information about race and income was not collected in the Danish survey. Further details on the sample characteristics are presented in Table 1.

**Table 1:** Participants’ characteristics, awareness, past experiences, and attitudes.

Variables		All participants (N=1600), n (%)	Participants in Brazil (n=1017), n (%)	Participants in Denmark (n=583), n (%)
**Age (years)**				
	18-27	933 (58.31)	613 (60.28)	320 (54.89)
	28-37	459 (28.69)	273 (26.84)	186 (31.90)
	38-47	105 (6.56)	80 (7.87)	25 (4.29)
	48-57	64 (4.00)	38 (3.74)	26 (4.46)
	>57	32 (2.00)	9 (0.88)	23 (3.95)
	Prefer not to say	7 (0.44)	4 (0.39)	3 (0.51)
**Gender**				
	Female	891 (55.69)	606 (59.59)	285 (48.89)
	Male	682 (42.63)	399 (39.23)	283 (48.54)
	Nonbinary	9 (0.56)	5 (0.49)	4 (0.69)
	Prefer not to say	18 (1.13)	7 (0.69)	11 (1.89)
**Self-reported race**				
	White	—^a^	555 (54.57)	—
	Black or Brown	—	439 (43.17)	—
	Yellow	—	3 (0.29)	—
	Indigenous	—	2 (0.20)	—
	Prefer not to say	—	18 (1.77)	—
**Household income (monthly)^b^**				
	Less or equal to 1 minimum salary	—	114 (11.21)	—
	Between 1 and 3 minimum salaries	—	340 (33.43)	—
	Between 3 and 5 minimum salaries	—	399 (39.23)	—
	Higher or equal to 5 minimum salaries	—	113 (11.11)	—
	Prefer not to say	—	51 (5.01)	—
**Education**				
	Less than secondary education	1 (0.06)	1 (0.10)	0 (0)
	Currently on higher education	595 (37.19)	479 (47.10)	116 (19.90)
	Higher education degree completed	992 (62.00)	527 (51.82)	465 (79.76)
	Prefer not to say	12 (0.75)	10 (0.98)	2 (0.34)
**Digital devices owned**				
	Smartphone(s)	1562 (97.63)	994 (97.74)	568 (97.43)
	Computer(s)	1537 (96.06)	962 (94.59)	575 (98.63)
	Tablet(s)	399 (24.94)	194 (19.08)	205 (35.16)
	Smartwatch(es)	267 (16.69)	145 (14.26)	122 (20.93)
	Smarthome assistant(s)	132 (8.25)	59 (5.80)	73 (12.52)
**Number of digital device types owned**				
	0	5 (0.31)	5 (0.49)	0 (0)
	1	64 (4.00)	50 (4.92)	14 (2.40)
	2	943 (58.94)	666 (65.49)	277 (47.51)
	3	437 (27.31)	227 (22.32)	210 (36.02)
	4	119 (7.44)	54 (5.31)	65 (11.15)
	5 or more	32 (2.00)	15 (1.47)	17 (2.92)
**Current health status**				
	Poor	30 (1.88)	16 (1.57)	14 (2.40)
	Fair	157 (9.81)	119 (11.70)	38 (6.52)
	Good	513 (32.06)	355 (34.91)	158 (27.10)
	Very good	666 (41.63)	406 (39.92)	260 (44.60)
	Excellent	228 (14.25)	117 (11.50)	111 (19.04)
	Prefer not to say	6 (0.38)	4 (0.39)	2 (0.34)
**Interest in health topics**				
	Not interested	39 (2.44)	21 (2.06)	18 (3.09)
	Slightly interested	471 (29.44)	247 (24.29)	224 (38.42)
	Moderately interested	124 (7.75)	72 (7.08)	52 (8.92)
	Very interested	559 (34.94)	340 (33.43)	219 (37.56)
	Extremely interested	405 (25.31)	336 (33.04)	69 (11.84)
	Prefer not to say	2 (0.13)	1 (0.10)	1 (0.17)
**Previous participation in a health research study**				
	No	815 (50.94)	440 (43.26)	375 (64.32)
	Yes	763 (47.69)	567 (55.75)	196 (33.62)
	Prefer not to say	22 (1.38)	10 (0.98)	12 (2.06)
**Awareness of examples of research data repositories**				
	No	884 (55.25)	528 (51.92)	356 (61.06)
	Yes	459 (28.69)	330 (32.45)	129 (22.13)
	Not sure	245 (15.31)	152 (14.95)	93 (15.95)
	Prefer not to say	12 (0.75)	7 (0.69)	5 (0.86)
**Perception of digital health data repositories**				
	Positive	1339 (83.69)	927 (91.15)	412 (70.67)
	Indifferent	188 (11.75)	53 (5.21)	135 (23.16)
	Negative	45 (2.81)	19 (1.87)	26 (4.46)
	Prefer not to say	28 (1.75)	18 (1.77)	10 (1.72)

^a^Data not collected.

^b^Ranges between 1 (1040 Brazilian Reais or US $190) and 5 (5200 Brazilian Reais or US $950).

### Previous Participation, Awareness of Examples, and Current Attitude

Around half of the respondents participated in a health research study before (763/1600, 47.69%), and those who participated are more likely to have a moderate to high interest in health topics (OR 2.35, 95% CI 1.88-2.93; *P*<.001). By contrast, only a minority are aware of research data repository examples (459/1600, 28.69%). Those aware of examples are more likely to have a moderate to high interest in health topics (OR 3.02, 95% CI 2.30-3.96; *P*<.001) and to have been participants in previous health studies (OR 3.36, 95% CI 2.66-4.23; *P*<.001). In addition, most participants have a positive perception of health research data repositories (1339/1600, 83.69%), and those who have a positive perception are more likely to be aware of examples of research data repositories (OR 3.26, 95% CI 2.17-4.90; *P*<.001). Further details on the frequency distribution for these variables are shown in Table 1.

Results from a binary logistic regression show that interest in health topics (OR 1.49, 95% CI 1.10-2.02; *P*=.01), previous participation in health research studies (OR 1.70, 95% CI 1.24-2.35; *P*=.001), and awareness of examples of existing repositories (OR 2.78, 95% CI 1.83-4.38; *P*<.001) are significant factors influencing participants’ current perception of digital health research repositories. See the results of the binary logistic regression in Table 2.

**Table 2:** Binary logistic regression model for the current perception of digital health data repositories (base: not positive perception).

Factors for current perception digital health data repositories (base: not positive perception)		Estimate (B)	Standard error B	*P* value	Odds ratio (95% CI)
**Age (base: above 27)**					
	Below 27 years	0.10	0.17	.56	1.11 (0.79-1.55)
**Gender (base: not female)**					
	Female	−0.05	0.15	.73	0.95 (0.70-1.28)
**Education (base: no university degree)**					
	With university degree	−0.29	0.17	.10	0.75 (0.52-1.06)
**Device ownership (base: less than 2 device types)**					
	Owns more than 2 types	−0.26	0.15	.07	0.76 (0.56-1.04)
**Health status (base: poor or fair health)**					
	Good, very good, or excellent health	0.06	0.23	.79	0.94 (0.57-1.74)
**Interest in health (base: none or slight interest)**					
	Moderate to extreme interest	0.39	0.15	.01	1.49 (1.10-2.02)
**Participation in health study (base: no past participation)**					
	Participated in a health study	0.53	0.16	.001	1.70 (1.24-2.35)
**Awareness of an example (base: no awareness or not sure)**					
	Aware of an example of repository	1.02	0.22	<.001	2.78 (1.83-4.38)

### Motivations to Participate

**Figure 1:**
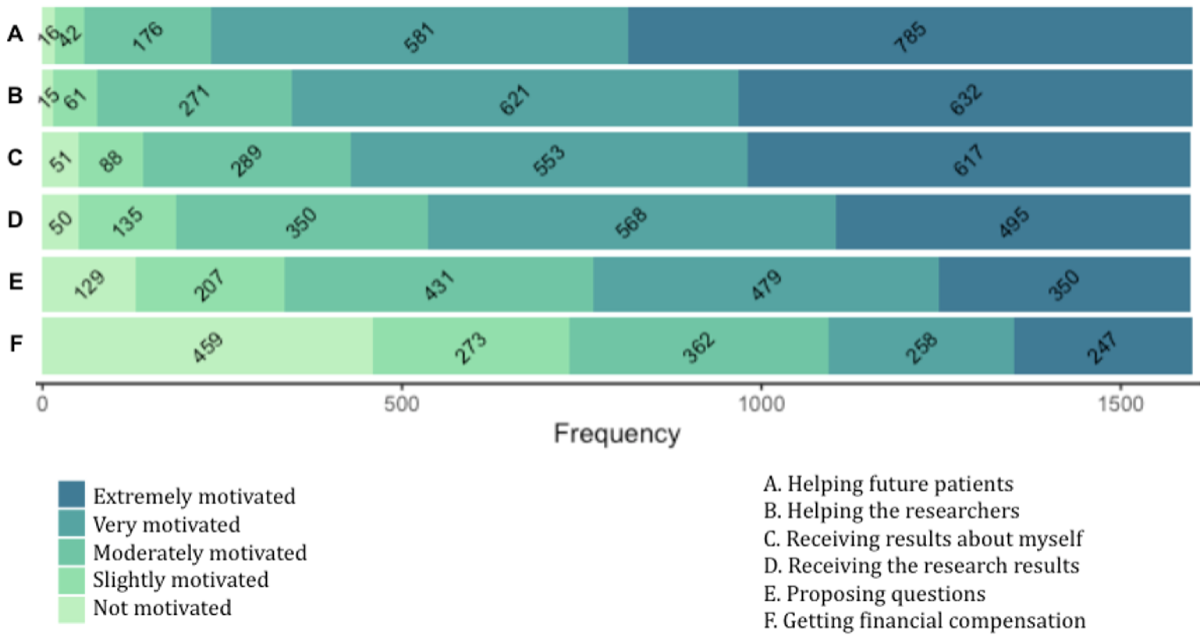
Bar chart displaying the distribution of answers for each motivation source.

The majority of participants feel very or extremely motivated by helping future patients (1366/1600, 85.38%), helping researchers (1253/1600, 78.31%), receiving results about themselves (1170/1600, 73.13%), and receiving the results of the research (1063/1600, 66.44%). In addition, being provided with the possibility of suggesting research questions to be investigated is very or extremely motivating for more respondents (829/1600, 51.81%) than receiving financial compensation (505/1600, 31.56%), which is not motivating for 28.69% (459/1600). Multimedia Appendix 2 shows the entire distribution of responses, and Figure 1 displays this information as stacked bar charts.

Those who have a positive perception about health data repositories are more likely to be moderately, very, or extremely motivated by 5 out of 6 motivation sources: helping future patients (OR 9.44, 95% CI 5.43-16.40; *P*<.001), helping researchers (OR 5.74, 95% CI 3.56-9.25; *P*<.001), receiving results about themselves (OR 4.12, 95% CI 2.82-6.03; *P*<.001), receiving results of the research (OR 4.15, 95% CI 2.94-5.85; *P*<.001), and proposing questions to be investigated (OR 3.46, 95% CI 2.57-4.66; *P*<.001). Those moderately, very, or extremely interested in health topics are more likely to be moderately, very, or extremely motivated by receiving results of the research (OR 2.25, 95% CI 1.65-3.06; *P*<.001) and proposing questions to be investigated (OR 2.53, 95% CI 1.97-3.24; *P*<.001). The youngest segment (18-27 years old) is more likely to feel moderately, very, or extremely motivated to receive financial compensation (OR 1.92, 95% CI 1.57-2.35; *P*<.001).

### Reasons for Concern

**Figure 2:**
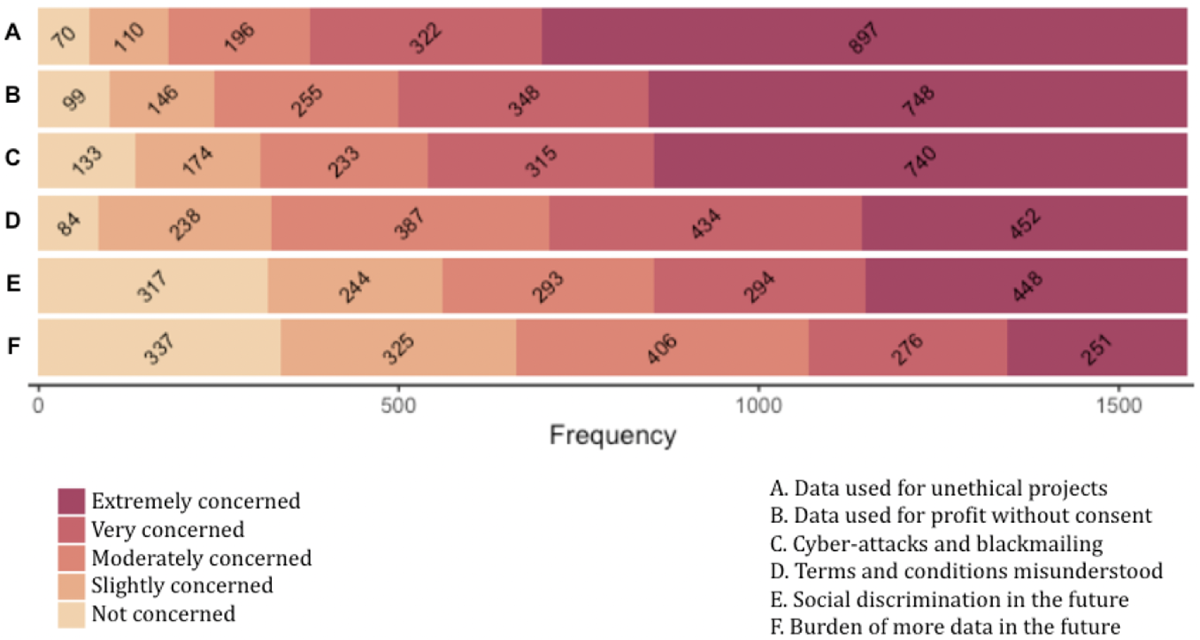
Bar chart displaying the distribution of answers for each reason for concern.

The vast majority of participants feel very or extremely concerned about having their data used for unethical projects (1219/1600, 76.19%) and profit making without their consent (1096/1600, 68.50%). In addition, the risk of becoming vulnerable to cyberattacks and blackmail is very or extremely concerning for 65.94% (1055/1600); the possibility of not understanding terms and conditions for 55.38% (886/1600); and the fear of being socially discriminated for 46.38% (742/1600). By contrast, not as many participants feel very or extremely concerned about the burden of being asked to share more data in the future (527/1600, 32.94%). Multimedia Appendix 3 shows the entire distribution of responses, and Figure 2 displays this information in the form of a stacked bar chart.

### Willingness to Share Different Types of Data

Regarding the willingness to share specific data items, most participants feel uncomfortable or very uncomfortable sharing the content of texts and calls (1206/1600, 75.38%), while fewer participants feel uncomfortable or very uncomfortable sharing the frequency of texts and calls (706/1600, 44.13%). Places visited (864/1600, 54.00%) and apps used (775/1600, 48.44%) are perceived as uncomfortable or very uncomfortable data to share by many.

**Figure 3:**
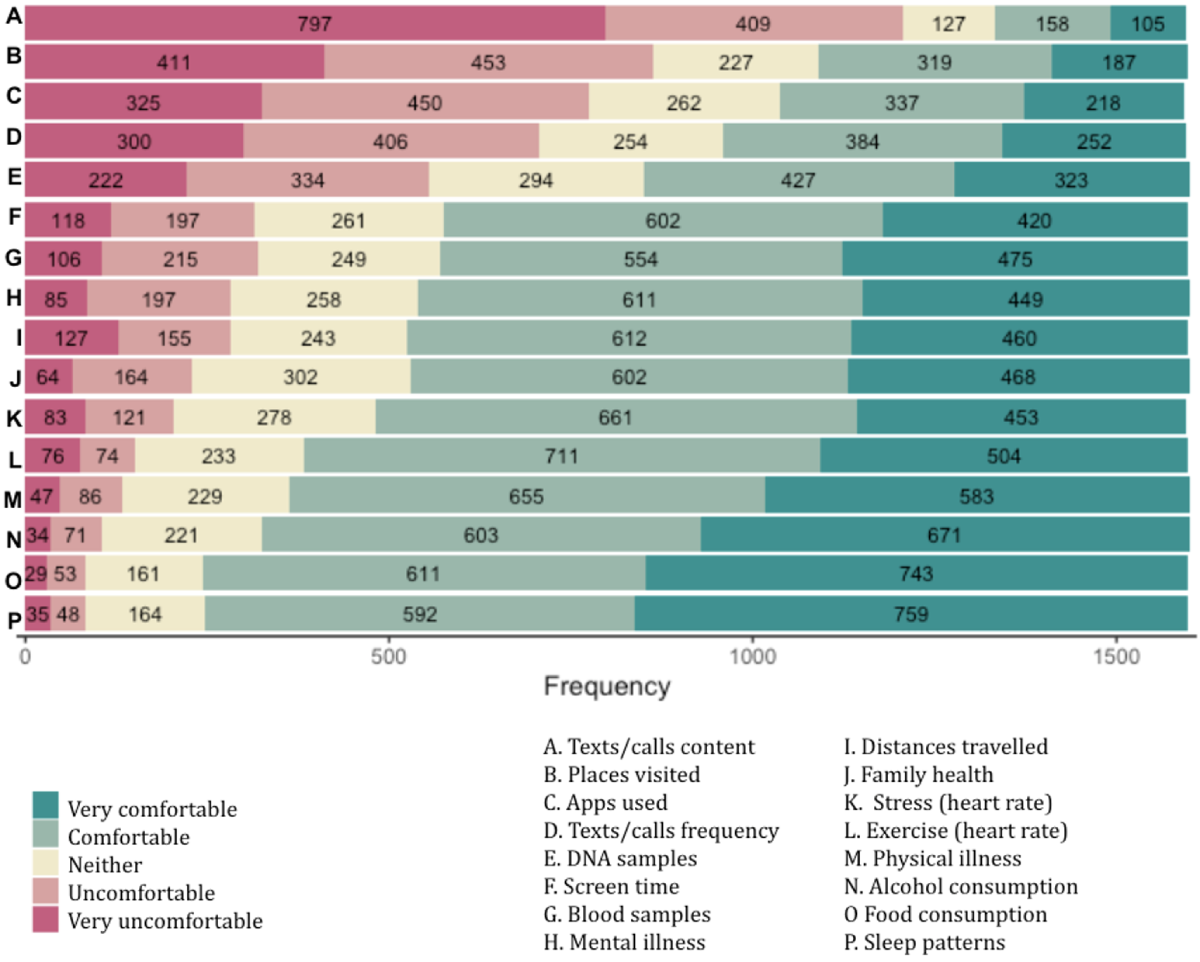
Bar chart displaying the distribution of willingness to share across different data types.

By contrast, most participants feel comfortable or very comfortable sharing sleeping patterns (1351/1600, 84.44%), food consumption (1354/1600, 84.63%), alcohol consumption (1274/1600, 79.63%), physical illness diagnosis (1238/1600, 77.38%), physical activity levels (1215/1600, 75.94%), stress levels (1114/1600, 69.63%), family health history (1070/1600, 66.88%), distances traveled (1072/1600, 67.00%), mental illness diagnosis (1060/1600, 66.25%), blood samples (1029/1600, 64.31%), DNA samples (750/1600, 46.88%), and screen time (1022/1600, 63.88%). Multimedia Appendices 4 and 5 show the full distribution of responses, and Figure 3 displays this information as stacked bar charts.

Those who have a positive perception about health research repositories are more likely to feel comfortable or very comfortable sharing 15 out of 16 data items: physical illness diagnosis (OR 3.84, 95% CI 2.87-5.15; *P*<.001), mental illness diagnosis (OR 3.44, 95% CI 2.59-4.59; *P*<.001), family health history (OR 3.45, 95% CI 2.59-4.59; *P*<.001), DNA samples (OR 2.51, 95% CI 1.85-3.41; *P*<.001), blood samples (OR 2.31, 95% CI 1.74-3.06; *P*<.001), food consumption (OR 4.15, 95% CI 3.01-5.70; *P*<.001), alcohol consumption (OR 3.25, 95% CI 2.41-4.40; *P*<.001), sleep (OR 3.85, 95% CI 2.80-5.30; *P*<.001), screen time (OR 3.17, 95% CI 2.38-4.22; *P*<.001), apps used (OR 2.09, 95% CI 1.50-2.91; *P*<.001), frequency of calls or texts (OR 2.07, 95% CI 1.51-2.83; *P*<.001), distances traveled per day (OR 3.34, 95% CI 2.51-4.45; *P*<.001), places visited (OR 2.77, 95% CI 1.91-4.00; *P*<.001), physical activity levels (OR 3.43, 95% CI 2.56-4.59; *P*<.001), and stress levels (OR 3.60, 95% CI 2.70-4.79; *P*<.001). However, no significant association was found between having a positive perception of digital health research repositories and feeling comfortable with sharing the content of calls and texts (*P*=.03).

Those moderately, very, or extremely concerned about being discriminated against are more likely to feel uncomfortable or very uncomfortable sharing data about mental illness diagnosis (OR 2.26, 95% CI 1.66-3.07; *P*<.001). Those uncomfortable or very uncomfortable sharing information about app usage are more likely to be moderately, very, or extremely concerned about data being used for profit (OR 2.57, 95% CI 1.91-3.46; *P*<.001) and not understanding terms and conditions (OR 2.22, 95% CI 1.71-2.87; *P*<.001). Those not motivated or only slightly motivated by receiving results about themselves are more likely to feel uncomfortable or very uncomfortable with sharing information about alcohol consumption (OR 5.76, 95% CI 3.63-9.13; *P*<.001), distances traveled per day (OR 3.31, 95% CI 2.29-4.80; *P*<.001), stress levels (OR 6.46, 95% CI 4.43-9.44; *P*<.001), and physical activity levels (OR 6.78, 95% CI 4.52-10.17; *P*<.001).

A small number of participants feel uncomfortable or very uncomfortable sharing any of the data items (94/1600, 5.88%). Those who feel uncomfortable or very uncomfortable sharing any data items are more likely to have a negative or indifferent perception about health research repositories (OR 3.91, 95% CI 2.49-6.14; *P*<.001). A binary logistic regression shows that age (OR 2.16, 95% CI 1.28-3.70; *P*=.004), digital device ownership (OR 1.90, 95% CI 1.14-3.26; *P*=.01), health status (OR 2.28, 95% CI 1.24-3.98; *P*=.01), and current attitude regarding digital health research repositories (OR 3.77, 95% CI 2.24-6.26; *P*<.001) are significant factors affecting participants’ willingness to share data with a health research repository. Table 3 shows the results of the binary logistic regression.

**Table 3:** Binary logistic regression model for willingness to share data with repositories for health research (base: unwilling to share any data).

Factors for willingness to share data (base: unwilling to share any)		Estimate (B)	Standard error B	*P* value	Odds ratio (95% CI)
**Age (base: above 27)**					
	Below 27 years	0.76	0.27	.004	2.16 (1.28-3.70)
**Gender (base: not female)**					
	Female	0.06	0.23	.78	1.06 (0.66-1.70)
**Education (base: no university degree)**					
	With university degree	0.21	0.28	.45	1.24 (0.70-2.16)
**Device ownership (base: less than 2 device types)**					
	Owns more than 2 devices	0.64	0.26	.01	1.90 (1.14-3.26)
**Health status (base: poor or fair health)**					
	Good, very good, or excellent health	−0.82	0.29	.005	2.28 (1.24-3.98)
**Interest in health (base: no or slight interest)**					
	Moderate to extreme interest	−0.01	0.25	.95	0.99 (0.59-1.62)
**Participation in health study (base: no past participation)**					
	Participated in a health study	0.14	0.24	.55	1.16 (0.71-1.90)
**Awareness of an example (base: no awareness)**					
	Aware of an example of repository	−0.12	0.28	.65	0.88 (0.51-1.56)
**Current perception (base: negative or indifferent)**					
	Positive current perception	1.32	0.26	<.001	3.77 (2.24-6.26)

### Preferred Access Control Options

**Figure 4:**
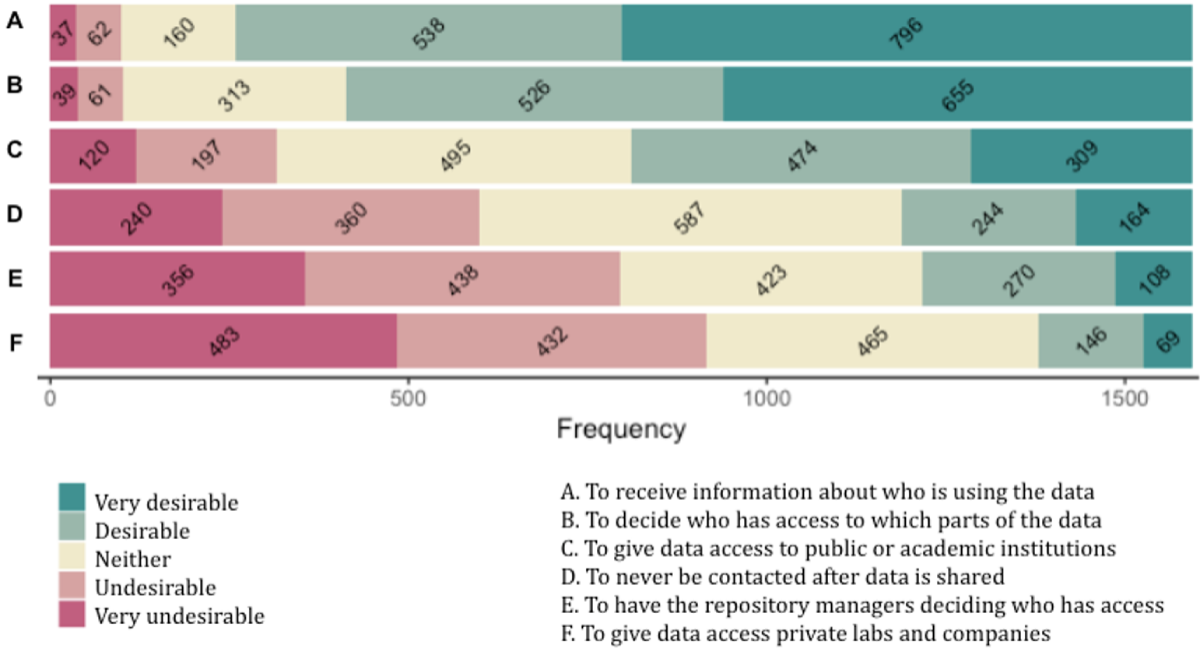
Bar chart displaying the distribution of answers for each access control option.

After collecting and sharing their data with a research platform, most participants find it desirable or very desirable to receive information about which projects access their data in the future (1334/1600, 83.38%). The majority also find it desirable or very desirable to decide who gets access to which parts of their data (1181/1600, 73.81%). By contrast, not being contacted is desirable or very desirable to only 25.50% of participants (408/1600), and the option to allow the owners of the repositories to decide who can access the data is desirable or very desirable only to 23.63% (378/1600). Finally, allowing public or academic institutions to access the data is desirable or very desirable for 48.94% (783/1600), while allowing private laboratories and companies to obtain access is desirable or very desirable to only 13.44% (215/1600). Multimedia Appendix 6 shows the entire distribution of responses, and Figure 4 displays this information as stacked bar charts.

Those who find it is desirable or very desirable to be informed about who is using their data are more likely to have a positive perception of health data repositories (OR 2.45, 95% CI 1.77-3.39; *P*<.001). Those moderately, very, or extremely concerned about data being used for unethical projects are more likely to find it desirable or very desirable to have control over how their data are used (OR 2.45, 95% CI 1.80-3.42; *P*<.001) and to be informed about it (OR 3.09, 95% CI 2.18-4.37; *P*<.001). Those moderately, very, or extremely concerned about data being used for profit are more likely to find it undesirable or very undesirable to have private laboratories and companies access their data (OR 2.24, 95% CI 1.69-2.96; *P*<.001).

### Cross-country Analysis

The sample comprises 1017 respondents living in Brazil (1017/1600, 63.56%) and 583 living in Denmark (583/1600, 36.44%). The demographics of the participants residing in these 2 countries do not differ substantially, except for a higher percentage of female respondents and a lower percentage of respondents who completed a higher education degree within the Brazilian sample (Table 1). The Brazilian sample also has a higher percentage of extremely interested individuals in health topics than the Danish sample (Table 1).

Around half of the participants in Brazil participated in health research previously (567/1017, 55.75%), in contrast to a minority of the participants in Denmark (196/583, 33.62%). Similarly, the vast majority of participants from the Brazilian sample have a positive initial impression of health data repositories (927/1017, 91.15%), in contrast to a smaller majority of participants in Denmark (412/583, 70.67%). See Table 1 for complete information regarding these differences.

The majority of participants from both countries are highly motivated and concerned by similar sources of motivation and concerns; however, there are differences in the magnitude of the motivation and concern levels reported by those residing in Brazil and Denmark. The Brazilian sample is more likely to rate themselves as moderately, very, or extremely motivated by receiving results about themselves (OR 6.35, 95% CI 4.25-9.50; *P*<.001), proposing questions to be investigated (OR 6.08, 95% CI 4.67-7.91; *P*<.001), receiving results of the research (OR 4.13, 95% CI 2.98-5.72; *P*<.001), and helping the researchers (OR 3.36, 95% CI 2.07-5.44; *P*<.001). The Brazilian sample is also more likely to rate levels of concerns as moderately, very, or extremely concerning for all of the options listed: having data used for unethical projects (OR 5.44, 95% CI 3.86-7.66; *P*<.001), becoming vulnerable to cyberattacks and blackmail (OR 3.88, 95% CI 2.99-5.04; *P*<.001), having data used for profit without consent (OR 3.68, 95% CI 2.77-4.89; *P*<.001), being asked to provide more data (OR 3.28, 95% CI 2.65-4.06; *P*<.001), agreeing to terms without understanding them (OR 2.29, 95% CI 1.79-2.94; *P*<.001), and being socially discriminated against (OR 2.10, 95% CI 1.70-2.60; *P*<.001). Multimedia Appendices 2 and 3 show the frequency distribution of answers regarding motivations and concerns according to country of residence.

When it comes to access preferences, important differences arise between the 2 countries. The Brazilian sample is more likely to find it desirable or very desirable to receive information about who is using the data (OR 5.51, 95% CI 4.12-7.37; *P*<.001). By contrast, the Danish sample is more likely to find it desirable or very desirable never to be contacted (OR 3.63, 95% CI 2.87-4.60; *P*<.001), to have the repository managers decide who can obtain access (OR 2.84, 95% CI 2.24-3.60; *P*<.001), and to allow private organizations (OR 3.73, 95% CI 2.77-5.04; *P*<.001) and public institutions access the data (OR 4.51, 95% CI 3.61-5.63; *P*<.001). Multimedia Appendices 4-6 show the frequency distribution of answers regarding access control preferences and willingness to share according to country of residence.

In summary, the vast majority of the participants residing in Brazil have a positive attitude regarding the idea of health data repositories. These findings are further endorsed by the Brazilian sample reporting higher motivation to help the researchers and willingness to share several data types. However, those residing in Brazil are also more likely to be strongly concerned about all of the potential negative consequences. The Brazilian sample is also more likely to find it desirable to keep the control and be informed about the use of the shared data, rather than never being contacted, delegating control to repository owners, or allowing both private companies and public institutions to get access.

## Discussion

### Enablers for Acceptance

Our survey contributes novel empirical insights regarding an extensive set of factors contributing to the acceptance of repositories storing biosamples, health records, and digital data sources for observational research. Previous research suggests that individuals may view some loss of privacy as worthwhile to advance medical research and benefit future generations [4,33], with altruism being a strong incentive for participation in clinical studies [50,67]. Aligned with such previous research, we found that helping future patients and researchers is indeed a powerful source of motivation across our sample, with most participants also feeling very motivated by the prospect of being updated about research outcomes. Furthermore, our findings show that those who do not feel motivated by helping future patients and researchers are more likely to be unwilling to share data, highlighting the critical role of altruism in this context.

Participants are also motivated by learning about their health through the data they provide, aligning with past research [68]. By contrast, our findings indicate that financial compensation may not be a more decisive factor than other sources of motivation. For instance, being invited to suggest research questions for a project strongly motivates more participants than financial compensation. However, consistent with previous studies [41,50], the youngest participants in our sample are more likely to be motivated to share health data in exchange for financial benefits. Such observations reinforce the importance of providing both societal and individual benefits to accommodate different preferences.

Another essential enabler for acceptance is individuals’ current perception of the idea of health research data repositories. In our survey, a positive perception appears to be associated with higher levels of motivation to help patients and researchers, and those who have a positive perception are also more likely to feel comfortable sharing 15 out of 16 data items. These results confirm past research highlighting that a positive opinion about biomedical research can predict willingness to participate [33], and attitudes about health care interventions can predict patient acceptance [56]. We also extend previous findings from other contexts [34,43,55] by providing evidence about key factors that can affect individuals’ perceptions of digital health research repositories, emphasizing the critical role that positive past experiences and personal interests have in enabling favorable attitudes.

Regarding cross-country differences, the vast majority of our participants residing in Brazil have a positive perception of the idea of health data repositories, further demonstrated by their reported higher motivation to participate and higher willingness to share several data types. It could be speculated that such enthusiasm stems from the prospect of significantly improving an imperfect yet ubiquitous public health care system, which may become an essential enabler for acceptance as digital health emerges in Brazil [67]. By contrast, Denmark has a long history of using clinical databases and electronic health records for population-level clinical research [44]. This observation could explain why our sample residing in Denmark is more likely to find it desirable to allow repository owners to make decisions regarding access control, an arrangement already familiar to them, as the Danish public health authorities manage data use. These differences illustrate that acceptance depends not only on individual predispositions but also on broader sociocultural contexts [36].

### Barriers for Acceptance

In contrast to such enablers, our findings show that even though participation in research repositories might occur under the promise of sharing deidentified data, participants still report concerns. Our sample’s most substantial concern is the fear that their data will eventually be used for unethical research goals or profit without consent, which is a fear also reported by several previous studies [37,38,42]. Further aligned to previous research, the fear of cyberattacks or blackmail is considered very or extremely concerning to most of our participants [69-72]. Surprisingly, however, the fear of social discrimination is not as prevalent, contrasting a previous study’s claim that this might be a core reason behind privacy concerns [58]. It is also surprising that the fear of not fully understanding terms and conditions was a more significant concern for participants than the burden of providing more data, which contradicts previous findings from another study [4].

The predominant concern of data misuse may explain the preference for more restrictive access control options. Many of our participants report feeling comfortable sharing their data if the purpose is to protect the common good, but the same does not apply to the prospect of supporting others’ profit making, in alignment with previous research [36,44,73]. Related to this, the large majority of our sample want to receive information about the different projects using their data, and most also want to be deciding who can ultimately obtain access to their data, a finding which has been highlighted in other past studies [38,55,73-75]. By contrast, leaving this responsibility to repository owners is often not our participant’ preferred option, especially within the Brazilian sample. Furthermore, approaches such as notification-only and opt-out options have been considered less acceptable than reconsent [73,76], showing the importance of reconsidering usual consent practices.

Another barrier to participation is that willingness to share data depends strongly on the data type [32,47,67], even though there are divergent findings in the literature about which data types people feel most uncomfortable sharing [37,38,42,61-65]. For example, previous studies with young adults have observed a high willingness to donate DNA samples [33,34], but 2 extensive worldwide surveys have observed the opposite [36,44]. Our analysis indicates that when compared with behavioral indicators such as food consumption and sleeping patterns, DNA and blood samples are among the data types most uncomfortable to be shared.

However, even more so than DNA, participants in our study feel uncomfortable sharing passive mobile and wearable sensing data. Interestingly, these are data with the less obvious connection to health in a traditional sense. While the relationship between health and food consumption or sleep might be apparent to many people, the relevance of app use or social communication data may be less noticeable. Such observation is particularly relevant for behavioral health research contemplating passive data sources as a strategy to reduce the data collection burden for participants. Our results also add a more nuanced understanding of participants’ willingness to share data. We empirically demonstrate that participants feel uncomfortable sharing more detailed and revealing data sources, such as apps used, frequency of texts and calls, and places visited, compared with broader and less granular information such as screen time, the content of texts and calls, and distances traveled. These findings have important implications for health research studies that consider collecting high granularity information, especially when it comes to location and social communication.

Furthermore, sociodemographic factors have been emphasized by several past studies as possible barriers to the willingness to share data [35,36,39,43,45,54,74]. Our analysis shows that participants’ willingness to share data can be related to age, health status, and digital device ownership. However, contrary to previous studies, which observed that members of American ethnic groups other than White have higher odds of being unwilling to donate their DNA data [43,45], our study does not find a significant association between race and unwillingness to share. We also do not find significant associations between race and fear of discrimination [43,45] or desire to control data access [35]. However, our sample is in its vast majority young and educated, in contrast to these previous studies.

### Research Implications

The empirical findings discussed above provide the basis for a series of implications for community engagement, technology design, and policymaking. First, we found evidence that a lack of knowledge about health research may be a challenge for public acceptance, which points to the importance of broadening public awareness. For instance, education and familiarity-increasing programs can be possible community engagement approaches and strengthened relationships between potential participants, clinicians, and health research experts may be helpful during recruitment and beyond [40]. Regardless of the medium, participant information could include explanations about the collaborative nature of contemporary health research and why digital data sources extracted passively may be necessary for answering specific research questions. Given the factors found to motivate and demotivate data sharing, it may be necessary to explain the benefits of sharing data types where the direct connection to health is not immediately visible. Additionally, appropriate communication may help to emphasize the importance of data collection compliance to participants, especially when it comes to experience sampling and the provision of frequent self-reports [77].

Personal health informatics could also be considered to increase the appeal of and the motivation for participating. Given that data collection may require interactions with mobile and wearable devices, it is a natural step to also provide participants with personalized data visualizations and, potentially, digital health interventions. However, digital tools for personal health must consider how existing health care practices complement (or hinder) novel approaches [78-80]. Interface design should focus on suitably informing patients about how their data relate to their health to facilitate rather than replace efficient clinician–patient relationships. Above all, risks to individual well-being should be avoided, as an intense “datafication” of personal health standards might prove to be more harmful than beneficial [81]. For instance, our analysis shows that those uncomfortable with sharing alcohol consumption, levels of stress, and physical exercise are less likely to feel motivated by receiving results about themselves. Thus, any consideration of adding personal health informatics features to health research systems should be mindful of the preferences of each individual.

Furthermore, our analysis makes it clear that broader acceptance will be challenging to achieve if contributing to health research repositories demands that participants share every digital source of data [38]. Health research projects might need to acknowledge that certain personal information is associated with social stigma [82], which may compromise willingness to participate in research as a whole. For instance, we observe a strong association between fear of discrimination and unwillingness to share mental illness diagnoses. For this reason, health research should consider personal boundaries by allowing participants to opt-out from specific data collection types and decide which level of details are to be shared. Even if individuals do not exercise this right to choose, the option to safely do that without negative consequences may still enhance trust [83].

When it comes to access control options, our results show that participants would like to be informed about the different projects which may access their data and customize their consent. Even though granular data control options may reduce privacy concerns [84], broad consent models are still the most used approach in current health research platforms [32], which means that once participants provide their consent, they are usually not consulted about data reuse in the future. The conception of digital systems for continuous communication with participants could transform consent practices. For instance, research participants could be consulted about whether they would like to receive a request each time a new project wants to use their data. Access requests could include details about who benefits from the research outcomes and how organizations use any profit. The possibility of opting out from data sharing could also be provided. Beyond allowing participants to make choices about data access, participants could further contribute with questions to a research project, which is an interest identified in our survey and explored in other research platforms [22]. Nondigital approaches could also be considered (eg, phone calls, letters) for those who prefer or do not have access to digital devices. However, a challenge is how to help participants stay informed and control their data without making them overwhelmed [42].

As pervasive sensing technologies become more refined and widespread in health research, those proposing shared-access repositories for collecting, sharing, and using such sensing data will need to take responsibility for identifying risks and be accountable for consequences against participants’ best interests. Proactive legal and ethical guidelines are necessary, as current regulatory frameworks for digital health data sharing are relatively weak in some jurisdictions [44]. Likewise, regulatory board members and grant reviewers could evaluate how managers of digital health research repositories demonstrate awareness of ethical considerations and strategies to mitigate possible negative consequences of participation. For instance, being transparent about the trustworthiness of the technical infrastructures and governance arrangements of the platforms hosting the data is essential, even if it means acknowledging challenges [50]. Clear and understandable evidence of compliance with regulations may help diminish individuals’ reticence to share health data and increase public acceptance.

Finally, future developments should not ignore that without a diverse cohort providing data, research outcomes and benefits will be unevenly distributed [42,85]. Even though our sample, composed mainly of educated young individuals, does not show significant associations between race, income, and unwillingness to share data, other past studies have shown that these factors can be significant [43,45]. For this reason, communication efforts, interface design, and data sharing policies should be made accessible and inclusive by being mindful of language choices, cultural requirements, access costs, and participation demands (eg, owning and using smartphones and smartwatches). After all, strategies to increase acceptance should be motivated by research repository owners’ genuine desire to make data sharing fairer and more ethical.

### Limitations and Future Work

Based on our team experience, we suggest that similar surveys in the future should strive to focus recruitment efforts on racial, ethnic, gender, and disability minorities to achieve a higher representation from these groups. We also suggest that quantitative findings should be complemented with parallel qualitative investigations, to provide richer and subjective insight into justifications and reasonings behind responses. Another suggestion is to consider depicting data usage scenarios with illustrations, infographics, and narrative forms instead of purely descriptive texts.

In terms of methodological limitations, sampling bias is a common challenge of voluntary response samples, given that those who take the time to respond to online survey requests tend to have strong opinions compared with the rest of the population. Despite our efforts to use a diverse range of digital channels for recruitment, our sample is biased in terms of age and education level, partially due to the scope of the overarching project (mental health for young adults) and partially because the higher response rate stemmed from posts on university web portals. A constrained focus on specific population segments can be considered a strength, given that it allows the investigation of particular perspectives at a time; however, future work must seek to consider the perspectives of many other groups of individuals, especially underrepresented minorities.

Another methodological limitation is that, as an exploratory cross-sectional study, our results can only capture attitudes about hypothetical future participation. Thus, future work must consider evaluating participants’ perspectives while taking part in an actual digital health research repository initiative. In addition, factors such as the burden of continuous data collection might be better examined throughout actual participation.

Finally, it could be speculated that public attitudes may shift following the global experience of a public health emergency (COVID-19 pandemic). Given that this survey was conducted during the first waves of the pandemic, future work is still needed to evaluate further consequences of this unprecedented crisis in the long term. In particular, the impact of contact tracing apps and vaccination passports may prove significant when it comes to the acceptance of digital health data storage on a population level.

### Conclusion

This survey study reveals essential factors for potential acceptance and willingness to share personal data with a digital health research repository. In summary, most participants feel very motivated about helping future patients, helping researchers, and receiving results about their health; most also feel comfortable sharing data sources usually associated with health research, except DNA data. However, most respondents feel very concerned about the risk of cyberattacks, the possibility of data being used for unethical research goals or for-profit without consent, and the prospect of sharing personal sensing data, especially social communication and location. The majority of participants find it desirable to receive information about which projects access their data and would like to be able to decide who gets access to which parts of their data.

The analysis of such a large spectrum of variables and their relationships provides a strong foundation for suggesting implications for future developments. The implications discussed include to disseminate knowledge about health research; to value the role of transparency for trust development; to engage participants with the research process and their health management; to allow flexible and customizable data sharing; and to align policies and regulations with ethical considerations. Providing valuable benefits for individuals and reducing the risks involved in participation are essential requirements in this context, and by recognizing differences between groups, it is possible to better understand and respond to individual views and expectations.

## Appendix

Multimedia Appendix 1 Questionnaire applied to participants (survey questions).

Multimedia Appendix 2 Distribution of answers for the question: “How motivated do you feel by the following?”.

Multimedia Appendix 3 Distribution of answers for the question: “How concerned are you about the following?”.

Multimedia Appendix 4 Distribution of answers for the question: “How comfortable do you feel about sharing the following data items (collected through questionnaires and surveys)?”.

Multimedia Appendix 5 Distribution of answers for the question: “How comfortable do you feel about sharing the following data items (collected through mobile and wearable sensing)?”.

Multimedia Appendix 6 Distribution of answers for the question: “How desirable are the following options?”.

## Abbreviations

DTU: Technical University of Denmark
GDPR: General Data Protection Regulation
