# Supplementary material for: Public Attitudes to Digital Health Research Repositories: Cross-sectional International Survey
Source: J Med Internet Res. 2021 Oct 29;23(10):e31294. doi: 10.2196/31294 (PMC8590194; doi:10.2196/31294)
Supplement: Multimedia Appendix 1 [file jmir_v23i10e31294_app1.pdf]

Please answer our survey and express your opinion about data sharing for health research. We want to hear from you to understand your boundaries and preferences.

Questions should take 10 minutes only. We hope you can help!

Section A: Informed Consent

We are a group of researchers from the Copenhagen Center for Health Technology (CACHET). Before we start collecting data, we need to be sure you read, understand and agree with the following:

What is the purpose of this survey? To understand people's opinions about health research for a university/academic project.

Which data will be collected? Voluntary demographics (e.g., age, gender), opinions about research and preferences about data sharing.

Will this data be linked to me personally? No, this survey is anonymous: no personally identifiable data is collected (e.g., name, e-mail, IP address, geo-location).

How will data be processed? Statistical analysis will be conducted by the principal researcher for scientific reports, which will only contain aggregate results.

How will data be stored? The anonymous answers will be stored at a secured repository hosted at the university until the end of the project (October 2021).

How can I delete my data? You can click on Exit and clear survey on the top right of the page or contact us with a unique ID code which you will create.

How to contact us? - Responsible researcher: PhD candidate, Giovanna Vilaza: gnvi@dtu.dk - Director of the CACHET Research Centre: Prof Dr Jakob Bardram: jakba@dtu.dk - Data protection officer at DTU, Ane Sandager: anesa@dtu.dk

A1. Do you confirm that:
- you are more than 18 years old;
- you have read and understood the information above; - and you voluntarily agree to participate in this survey?

Yes ☐

No ☐

Section B: Demographics

B1. What is your age group?

- 18-27 ☐
- 28-37 ☐
- 38-47 ☐
- 48-57 ☐
- Above 57 ☐
- Prefer not to say ☐

B2. What is your gender?

- Female ☐
- Male ☐
- Non-binary ☐
- Prefer not to say ☐
- Prefer to self-describe ☐

Prefer to self-describe

[ ]

B3. What is your education level?

- Less than High School/Secondary School ☐
- Completed High School/Secondary School ☐
- Completed a Bachelor's degree/Undergraduate ☐
- Completed a Master's degree/Graduate ☐
- Completed a PhD degree or above ☐
- Prefer not to say ☐
- Other ☐

Other

[ ]

B4. Which of these digital devices do you have and use?

- [ ] Computer (desktop, laptop)
- [ ] Tablet (iPad)
- [ ] Smartphone (Samsung, iPhone)
- [ ] Wrist wearables (Apple Watch, Garmin, Fitbit, Polar)
- [ ] Smart home devices (Alexa, Google Home, Hive, Philips Hue)
- [ ] Other

Other

B5. In which country do you currently live?

- [ ] Afghanistan
- [ ] Albania
- [ ] Algeria
- [ ] Andorra
- [ ] Angola
- [ ] Antigua and Barbuda
- [ ] Argentina
- [ ] Armenia
- [ ] Australia
- [ ] Austria
- [ ] Azerbaijan
- [ ] Bahamas
- [ ] Bahrain
- [ ] Bangladesh
- [ ] Barbados
- [ ] Belarus
- [ ] Belgium
- [ ] Belize

Benin

Bhutan

Bolivia (Plurinational State of)

Bosnia and Herzegovina

Botswana

Brazil

Brunei Darussalam

Bulgaria

Burkina Faso

Burundi

Cabo Verde

Cambodia

Cameroon

Canada

Central African Republic

Chad

Chile

China

Colombia

Comoros

Congo

Cook Islands

Costa Rica

Côte d'Ivoire

Croatia

Cuba

Cyprus

Czech Republic

Democratic People's Republic of Korea

- [ ] Congo
- [ ] Denmark
- [ ] Djibouti
- [ ] Dominica
- [ ] Dominican Republic
- [ ] Ecuador
- [ ] Egypt
- [ ] El Salvador
- [ ] Equatorial Guinea
- [ ] Eritrea
- [ ] Estonia
- [ ] Ethiopia
- [ ] Fiji
- [ ] Finland
- [ ] France
- [ ] Gabon
- [ ] Gambia
- [ ] Georgia
- [ ] Germany
- [ ] Ghana
- [ ] Greece
- [ ] Grenada
- [ ] Guatemala
- [ ] Guinea
- [ ] Guinea-Bissau
- [ ] Guyana
- [ ] Haiti
- [ ] Honduras
- [ ] Hungary

Iceland

India

Indonesia

Iran (Islamic Republic of)

Iraq

Ireland

Israel

Italy

Jamaica

Japan

Jordan

Kazakhstan

Kenya

Kiribati

Kuwait

Kyrgyzstan

Lao People's Democratic Republic

Latvia

Lebanon

Lesotho

Liberia

Libya

Lithuania

Luxembourg

Madagascar

Malawi

Malaysia

Maldives

Mali

Malta

Marshall Islands

Mauritania

Mauritius

Mexico

Micronesia (Federated States of)

Monaco

Mongolia

Montenegro

Morocco

Mozambique

Myanmar

Namibia

Nauru

Nepal

Netherlands

New Zealand

Nicaragua

Niger

Nigeria

Niue

Norway

Oman

Pakistan

Palau

Panama

Papua New Guinea

Paraguay

Peru

Philippines

Poland

Portugal

Puerto Rico

Qatar

Republic of Korea

Republic of Moldova

Romania

Russian Federation

Rwanda

Saint Kitts and Nevis

Saint Lucia

Saint Vincent and the Grenadines

Samoa

San Marino

Sao Tome and Principe

Saudi Arabia

Senegal

Serbia

Seychelles

Sierra Leone

Singapore

Slovakia

Slovenia

Solomon Islands

Somalia

South Africa

South Sudan

Spain

Sri Lanka

Sudan

Suriname

Swaziland

Sweden

Switzerland

Syrian Arab Republic

Tajikistan

Thailand

The former Yugoslav Republic of Macedonia

Timor-Leste

Togo

Tokelau

Tonga

Trinidad and Tobago

Tunisia

Turkey

Turkmenistan

Tuvalu

Uganda

Ukraine

United Arab Emirates

the United Kingdom of Great Britain and Northern Ireland

United Republic of Tanzania

United States of America

Uruguay

Uzbekistan

Vanuatu

Venezuela (Bolivarian Republic of)

Viet Nam ☐

Yemen ☐

Zambia ☐

Zimbabwe ☐

Other ☐

Other

Section C: Health

C1. In general, would you say your health is:

Excellent ☐

Very good ☐

Good ☐

Fair ☐

Poor ☐

Prefer not to say ☐

C2. How interested are you in health-related topics?

Extremely interested ☐

Very interested ☐

Moderately interested ☐

Slightly interested ☐

Not interested ☐

Prefer not to say ☐

C3. Have you ever participated in a research study about health?

Yes ☐

No ☐

Prefer not to say ☐

Section D: Research data repository

The following definition is very important for the next questions. Please read it carefully:

A research data repository is an online database containing data collected in previous studies. De-identified or anonymous data is stored to be re-used in the future by multiple researchers.

D1. Do you know any example of a research data repository?

- Yes ☐
- No ☐
- Not sure ☐
- Prefer not to say ☐

D2. Your current opinion about this idea of data repositories for health research is:

- Positive ☐
- Negative ☐
- Indifferent ☐
- Prefer not to say ☐

Section E: Sharing your data with a repository (I)

How comfortable do you feel about having the following data stored in a research repository?

Please consider that any information shared is de-identified or anonymised before being analysed by researchers.

E1. Health questionnaires (online or in-person):

	Very unco mfortable	Uncomfort able	Neither	Comfortabl e	Very comfortabl e
Clinical diagnosis (physical)	☐	☐	☐	☐	☐
Clinical diagnosis (mental)	☐	☐	☐	☐	☐
Family health status	☐	☐	☐	☐	☐
DNA samples	☐	☐	☐	☐	☐
Food consumption	☐	☐	☐	☐	☐
Alcohol consumption	☐	☐	☐	☐	☐

	Very uncomfortable	Uncomfortable	Neither	Comfortable	Very comfortable
Sleep	☐	☐	☐	☐	☐
Blood samples	☐	☐	☐	☐	☐

Section F: Sharing your data with a repository (II)

How comfortable do you feel about having the following data stored in a research repository?

Please consider that any information shared is <u>de-identified or anonymised</u> before being analysed by researchers.

F1. Passive monitoring through a phone or wearable device:

	Very uncomfortable	Uncomfortable	Neither	Comfortable	Very comfortable
Screen time	☐	☐	☐	☐	☐
Apps used	☐	☐	☐	☐	☐
Frequency of social communication (calls/text)	☐	☐	☐	☐	☐
Content of social communication (calls/text)	☐	☐	☐	☐	☐
Distances travelled per day	☐	☐	☐	☐	☐
Places visited every day	☐	☐	☐	☐	☐
Physical activity levels (heart rate)	☐	☐	☐	☐	☐
Stress/emotional levels (heart rate)	☐	☐	☐	☐	☐

Section G: Motivations

G1. Still in the context of research data repositories, how motivated do you feel by the following reasons to contribute to one of them?

	Not motivated	Slightly motivated	Moderately motivated	Very motivated	Extremely motivated
Helping future patients	☐	☐	☐	☐	☐
Helping the researchers	☐	☐	☐	☐	☐
Receiving results about myself	☐	☐	☐	☐	☐
Getting financial compensation	☐	☐	☐	☐	☐

	Not motivated	Slightly motivated	Moderately motivated	Very motivated	Extremely motivated
Proposing questions to be investigated	☐	☐	☐	☐	☐
Receiving the results of the research	☐	☐	☐	☐	☐

Section H: Access options

H1. How desirable/undesirable do you think the following access options for a health research data repository are?

	Very undesirable	Undesirable	Neither	Desirable	Very desirable
To receive information about the projects using my data	☐	☐	☐	☐	☐
To not be contacted after I share my data	☐	☐	☐	☐	☐
To decide who can have access to which parts my data	☐	☐	☐	☐	☐
To have the repository managers deciding who can get access	☐	☐	☐	☐	☐
That public or academic institutions are eligible for access	☐	☐	☐	☐	☐
That private labs and companies are eligible for access	☐	☐	☐	☐	☐

Section I: Concerns

I1. How concerned do you feel about the following risks of having your health data stored in a research repository?

	Not concerned	Slightly concerned	Moderately concerned	Very concerned	Extremely concerned
Being vulnerable to cyber-attacks and blackmailing	☐	☐	☐	☐	☐
Having my data used for projects that I think are unethical	☐	☐	☐	☐	☐
Having my data used for profit without me knowing about it	☐	☐	☐	☐	☐
Being socially discriminated because of certain information I shared	☐	☐	☐	☐	☐
Agreeing with terms and conditions that I do not fully understand	☐	☐	☐	☐	☐
Being asked to provide more data in the future	☐	☐	☐	☐	☐

Section J: Feedback

J1. **Do you allow your anonymous answers for this survey to be stored in an open repository and be accessed by other researchers?**

Yes ☐

No ☐

J2. **This survey is anonymous, which makes it impossible for us to know which answer is yours. If later on you want to access your answers or have them deleted, please create an ID code for you now (e.g., hello123), and send it to us by e-mail:**

Contact: gnvi@dtu.dk or jakba@dtu.dk

J3. **Please, let us know if you have any comment or feedback about this survey:**

Thank you so much for your time and answer! We really appreciate that.

Do you want to receive the final survey results? Please contact: Giovanna Vilaza, PhD candidate, at gnvi@dtu.dk or Prof. Jakob Bardram, at jakba@dtu.dk
