# Supplementary material for: Public Attitudes to Digital Health Research Repositories: Cross-sectional International Survey
Source: J Med Internet Res. 2021 Oct 29;23(10):e31294. doi: 10.2196/31294 (PMC8590194; doi:10.2196/31294)
Supplement: Multimedia Appendix 2 [file jmir_v23i10e31294_app2.pdf]

Motivation sources		All participants, n (%)	Participants in Brazil, n (%)	Participants in Denmark, n (%)
**Helping future patients**				
	Not or slightly motivated	58 (3.62)	25 (2.45)	33 (5.66)
	Moderately to extremely motivated	1542 (96.75)	992 (97.5)	550 (94.3)
	Prefer not to say	0 (0)	0 (0)	0 (0)
**Helping the researchers**				
	Not or slightly motivated	76 (4.75)	27 (2.65)	59 (10.12)
	Moderately to extremely motivated	1524 (95.25)	990 (97.34)	534 (91.59)
	Prefer not to say	0 (0)	0 (0)	0 (0)
**Receiving results about myself**				
	Not or slightly motivated	139 (8.68)	34 (0.33)	105 (18.01)
	Moderately to extremely motivated	1459 (91.18)	982 (96.55)	477 (81.81)
	Prefer not to say	2 (0.125)	1 (0.09)	1 (0.17)
**Receiving the research results**				
	Not or slightly motivated	185 (11.56)	62 (6.09)	123 (21.09)
	Moderately to extremely motivated	1413 (88.31)	955 (93.9)	458 (78.55)
	Prefer not to say	2 (0.125)	0 (0)	2 (0.34)
**Proposing questions to be investigated**				
	Not or slightly motivated	336 (21.00)	102 (10.02)	234 (40.13)
	Moderately to extremely motivated	1260 (88.31)	915 (89.97)	345 (59.17)
	Prefer not to say	4 (0.25)	0 (0.00)	4 (0.68)
**Getting financial compensation**				
	Not or slightly motivated	732 (45.75)	457 (44.93)	275 (47.16)

Motivation sources		All participants, n (%)	Participants in Brazil, n (%)	Participants in Denmark, n (%)
	Moderately to extremely motivated	867 (54.18)	560 (55.06)	307 (52.65)
	Prefer not to say	1 (0.06)	0 (0)	1 (0.17)
