# Supplementary material for: Public Attitudes to Digital Health Research Repositories: Cross-sectional International Survey
Source: J Med Internet Res. 2021 Oct 29;23(10):e31294. doi: 10.2196/31294 (PMC8590194; doi:10.2196/31294)
Supplement: Multimedia Appendix 3 [file jmir_v23i10e31294_app3.pdf]

Reasons for concern		All participants, n (%)	Participants in Brazil, n (%)	Participants in Denmark, n (%)
**Data is used for research with unethical goals**				
	Not or slightly concerned	180 (11.25)	51 (5.01)	129 (22.12)
	Moderately to extremely concerned	1415 (88.43)	966 (94.98)	449 (77.01)
	Prefer not to say	5 (0.31)	0 (0)	5 (0.85)
**Data is used for profit without explicit consent**				
	Not or slightly concerned	245 (15.31)	91 (8.94)	154 (26.41)
	Moderately to extremely concerned	1351 (84.43)	926 (91.05)	425 (72.89)
	Prefer not to say	4 (0.25)	0 (0)	4 (0.68)
**Suffering from cyber-attacks and blackmail**				
	Not or slightly concerned	307 (19.18)	115 (11.3)	192 (32.93)
	Moderately to extremely concerned	1288 (80.50)	901 (88.59)	387 (66.38)
	Prefer not to say	5 (0.31)	1 (0.09)	4 (0.68)
**Agreeing with terms without fully understanding**				
	Not or slightly concerned	322 (20.12)	154 (15.14)	168 (28.81)
	Moderately to extremely concerned	1273 (79.56)	863 (84.85)	410 (70.32)
	Prefer not to say	5 (0.31)	0 (0)	5 (0.85)
**Being socially discriminated because of the data**				
	Not or slightly concerned	561 (35.06)	294 (28.9)	267 (45.79)
	Moderately to extremely concerned	1035 (64.68)	723 (71.09)	312 (53.51)
	Prefer not to say	4 (0.25)	0 (0)	4 (0.68)
**Being asked to provide more data in the future**				

Reasons for concern		All participants, n (%)	Participants in Brazil, n (%)	Participants in Denmark, n (%)
	Not or slightly concerned	662 (41.37)	316 (31.07)	346 (59.34)
	Moderately to extremely concerned	933 (58.31)	700 (68.82)	233 (39.96)
	Prefer not to say	5 (0.31)	1 (0.09)	4 (0.68)
