# Supplementary material for: Public Attitudes to Digital Health Research Repositories: Cross-sectional International Survey
Source: J Med Internet Res. 2021 Oct 29;23(10):e31294. doi: 10.2196/31294 (PMC8590194; doi:10.2196/31294)
Supplement: Multimedia Appendix 4 [file jmir_v23i10e31294_app4.pdf]

Willingness to share questionnaire and biospecimen data	All participants, n (%)	Participants in Brazil, n (%)	Participants in Denmark, n (%)
**Sleep patterns**			
Uncomfortable or very uncomfortable	83 (5.18)	38 (3.73)	45 (7.71)
Comfortable or very comfortable	1351 (84.43)	873 (85.84)	478 (81.98)
Neither uncomfortable nor comfortable	164 (10.25)	106 (10.42)	58 (9.94)
Prefer not to say	2 (0.12)	0 (0)	2 (0.34)
**Food consumption**			
Uncomfortable or very uncomfortable	82 (5.12)	40 (3.93)	42 (7.2)
Comfortable or very comfortable	1354 (84.62)	865 (85.05)	489 (83.87)
Neither uncomfortable nor comfortable	161 (10.06)	111 (10.91)	50 (8.57)
Prefer not to say	3 (0.18)	1 (0.09)	2 (0.34)
**Alcohol consumption**			
Uncomfortable or very uncomfortable	105 (6.56)	36 (3.53)	69 (11.83)
Comfortable or very comfortable	1274 (79.62)	817 (80.33)	457 (78.38)
Neither uncomfortable nor comfortable	221 (13.81)	164 (16.12)	57 (9.77)
Prefer not to say	0 (0)	0 (0)	0 (0)
**Clinical diagnosis (physical)**			
Uncomfortable or very uncomfortable	133 (8.31)	54 (5.3)	79 (13.55)
Comfortable or very comfortable	1238 (77.37)	800 (78.66)	438 (75.12)
Neither uncomfortable nor comfortable	229 (14.31)	163 (16.02)	66 (11.32)

Willingness to share questionnaire and biospecimen data		All participants, n (%)	Participants in Brazil, n (%)	Participants in Denmark, n (%)
	Prefer not to say	0 (0)	0 (0)	0 (0)
**Family health**				
	Uncomfortable or very uncomfortable	228 (14.25)	105 (10.32)	123 (21.09)
	Comfortable or very comfortable	1070 (66.87)	710 (69.81)	360 (61.74)
	Neither uncomfortable nor comfortable	302 (18.87)	202 (19.86)	100 (17.15)
	Prefer not to say	0 (0)	0 (0)	0 (0)
**Clinical diagnosis (mental)**				
	Uncomfortable or very uncomfortable	282 (17.62)	132 (12.97)	150 (25.72)
	Comfortable or very comfortable	1060 (66.25)	702 (69.02)	358 (61.4)
	Neither uncomfortable nor comfortable	258 (16.12)	183 (17.99)	75 (12.86)
	Prefer not to say	0 (0)	0 (0)	0 (0)
**Blood samples**				
	Uncomfortable or very uncomfortable	321 (20.06)	211 (20.74)	110 (18.86)
	Comfortable or very comfortable	1029 (64.31)	612 (60.17)	417 (71.52)
	Neither uncomfortable nor comfortable	249 (15.56)	194 (19.07)	55 (9.43)
	Prefer not to say	1 (0.06)	0 (0)	1 (0.17)
**DNA samples**				
	Uncomfortable or very uncomfortable	556 (34.75)	319 (31.36)	237 (40.65)
	Comfortable or very comfortable	750 (46.87)	488 (47.98)	262 (44.93)
	Neither uncomfortable nor comfortable	294 (18.37)	210 (20.64)	84 (14.4)

Willingness to share questionnaire and biospecimen data		All participants, n (%)	Participants in Brazil, n (%)	Participants in Denmark, n (%)
	Prefer not to say	0 (0)	0 (0)	0 (0)
