# Supplementary material for: Public Attitudes to Digital Health Research Repositories: Cross-sectional International Survey
Source: J Med Internet Res. 2021 Oct 29;23(10):e31294. doi: 10.2196/31294 (PMC8590194; doi:10.2196/31294)
Supplement: Multimedia Appendix 5 [file jmir_v23i10e31294_app5.pdf]

Willingness to share mobile and wearable sensing data		All participants, n (%)	Participants in Brazil, n (%)	Participants in Denmark, n (%)
**Physical activity levels (heart rate)**				
	Uncomfortable or very uncomfortable	150 (9.37)	66 (6.48)	84 (14.4)
	Comfortable or very comfortable	1215 (75.93)	786 (77.28)	429 (73.58)
	Neither uncomfortable nor comfortable	233 (14.56)	165 (16.22)	68 (11.66)
	Prefer not to say	2 (0.12)	0 (0)	2 (0.34)
**Stress/emotional levels (heart rate)**				
	Uncomfortable or very uncomfortable	204 (12.75)	82 (8.06)	122 (20.92)
	Comfortable or very comfortable	1114 (69.62)	735 (72.27)	379 (65)
	Neither uncomfortable nor comfortable	278 (17.37)	199 (19.56)	79 (13.55)
	Prefer not to say	4 (0.25)	1 (0.09)	3 (0.51)
**Distances travelled per day**				
	Uncomfortable or very uncomfortable	282 (17.62)	168 (16.51)	114 (19.55)
	Comfortable or very comfortable	1072 (67)	678 (66.66)	394 (67.58)
	Neither uncomfortable nor comfortable	243 (15.18)	171 (16.81)	72 (12.34)
	Prefer not to say	3 (0.18)	0 (0)	3 (0.51)
**Screen time**				
	Uncomfortable or very uncomfortable	315 (19.68)	189 (18.58)	126 (21.61)
	Comfortable or very comfortable	1022 (63.87)	644 (63.62)	378 (64.83)
	Neither uncomfortable nor comfortable	261 (16.31)	184 (18.09)	77 (13.2)

Willingness to share mobile and wearable sensing data	All participants, n (%)	Participants in Brazil, n (%)	Participants in Denmark, n (%)
Prefer not to say	2 (0.12)	0 (0)	2 (0.34)
**Frequency of social communication (calls/text)**			
Uncomfortable or very uncomfortable	706 (44.12)	479 (47.09)	227 (38.96)
Comfortable or very comfortable	636 (39.75)	353 (34.7)	283 (48.54)
Neither uncomfortable nor comfortable	254 (15.87)	185 (18.19)	69 (11.83)
Prefer not to say	4 (0.25)	0 (0)	4 (0.68)
**Apps used**			
Uncomfortable or very uncomfortable	775 (48.43)	534 (52.5)	241 (41.33)
Comfortable or very comfortable	555 (34.68)	312 (30.67)	243 (41.68)
Neither uncomfortable nor comfortable	262 (16.37)	167 (16.42)	95 (16.29)
Prefer not to say	8 (0.5)	4 (0.39)	4 (0.68)
**Places visited every day**			
Uncomfortable or very uncomfortable	864 (54)	534 (52.5)	330 (56.6)
Comfortable or very comfortable	503 (31.43)	330 (32.44)	176 (30.18)
Neither uncomfortable nor comfortable	227 (14.18)	153 (15.04)	74 (8.06)
Prefer not to say	3 (0.18)	0 (0)	3 (0.51)
**Content of social communication (calls/text)**			
Uncomfortable or very uncomfortable	1206 (75.37)	794 (78.07)	412 (70.66)
Comfortable or very comfortable	263 (16.43)	141 (13.86)	122 (20.92)
Neither uncomfortable nor comfortable	127 (7.93)	80 (7.86)	47 (8.06)

Willingness to share mobile and wearable sensing data	All participants, n (%)	Participants in Brazil, n (%)	Participants in Denmark, n (%)
Prefer not to say	4 (0.25)	2 (0.19)	2 (0.34)
