# Supplementary material for: Public Attitudes to Digital Health Research Repositories: Cross-sectional International Survey
Source: J Med Internet Res. 2021 Oct 29;23(10):e31294. doi: 10.2196/31294 (PMC8590194; doi:10.2196/31294)
Supplement: Multimedia Appendix 6 [file jmir_v23i10e31294_app6.pdf]

Desirability of access control options		All participants, n (%)	Participants in Brazil, n (%)	Participants in Denmark, n (%)
**To receive information about who is using my data**				
	Undesirable or very undesirable	99 (6.18)	28 (2.75)	71 (12.17)
	Desirable or very desirable	1334 (83.37)	939 (92.33)	395 (67.75)
	Neither undesirable nor desirable	160 (10)	10 (0.98)	110 (18.86)
	Prefer not to say	7 (0.43)	0 (0)	7 (1.20)
**To decide who can have access to which parts my data**				
	Undesirable or very undesirable	100 (6.25)	44 (4.32)	56 (9.6)
	Desirable or very desirable	1181 (73.81)	785 (73.72)	396 (67.92)
	Neither undesirable nor desirable	313 (19.56)	188 (18.48)	125 (21.44)
	Prefer not to say	6 (0.37)	0 (0)	6 (1.02)
**That public or academic institutions can get access**				
	Undesirable or very undesirable	317 (19.81)	263 (25.86)	54 (9.26)
	Desirable or very desirable	783 (48.93)	367 (36.08)	416 (71.35)
	Neither undesirable nor desirable	495 (30.93)	386 (37.95)	109 (18.69)
	Prefer not to say	5 (0.31)	1 (0.09)	4 (0.68)
**To never be contacted after I share my data**				
	Undesirable or very undesirable	600 (37.5)	455 (44.73)	145 (24.87)
	Desirable or very desirable	408 (25.5)	167 (16.42)	241 (41.33)
	Neither undesirable nor desirable	587 (36.68)	395 (38.83)	192 (32.93)
	Prefer not to say	5 (0.31)	0 (0)	5 (0.85)

Desirability of access control options		All participants, n (%)	Participants in Brazil, n (%)	Participants in Denmark, n (%)
**To have the repository managers deciding who can get access**				
	Undesirable or very undesirable	794 (49.62)	589 (57.91)	205 (35.16)
	Desirable or very desirable	378 (23.62)	169 (16.61)	209 (35.84)
	Neither undesirable nor desirable	423 (26.43)	259 (25.46)	164 (28.13)
	Prefer not to say	5 (0.31)	0 (0)	5 (0.85)
**That private labs and companies can get access**				
	Undesirable or very undesirable	915 (57.18)	659 (64.79)	256 (43.91)
	Desirable or very desirable	215 (13.43)	78 (7.66)	137 (23.49)
	Neither undesirable nor desirable	465 (29.06)	280 (27.53)	185 (31.73)
	Prefer not to say	5 (0.31)	0 (0)	5 (0.85)
